# Neurofibroma of the bile duct: a rare cause of obstructive jaundice

**DOI:** 10.1308/003588413X13511609955931

**Published:** 2013-03

**Authors:** A De Rosa, D Gomez, AM Zaitoun, IC Cameron

**Affiliations:** Nottingham University Hospitals NHS Trust,UK

**Keywords:** Neurofibroma, Jaundice

## Abstract

Neurofibromas of the common bile duct are extremely rare. The lack of specific clinical or radiological features makes preoperative diagnosis in the absence of histology difficult. We report the case of a female patient who presented with obstructive jaundice and evidence of a common bile duct stricture on imaging. She underwent an exploratory laparotomy, and intraoperative frozen section confirmed clear margins and a benign lesion. Excision of the extrahepatic bile duct and A Roux-en-Y hepaticojejunostomy was performed. We discuss the clinical features and management of neurofibromas of the bile duct in light of the literature.

Neurofibromas of the common bile duct are extremely rare. The lack of specific clinical or radiological features makes preoperative diagnosis in the absence of histology difficult.^1^ In addition, patients tend to present with clinical symptoms and signs suggestive of underlying malignancy. We report a case of neurofibroma of the common bile duct, and discuss the surgical treatment and histopathological findings.

## Case history

**Figure 1:**
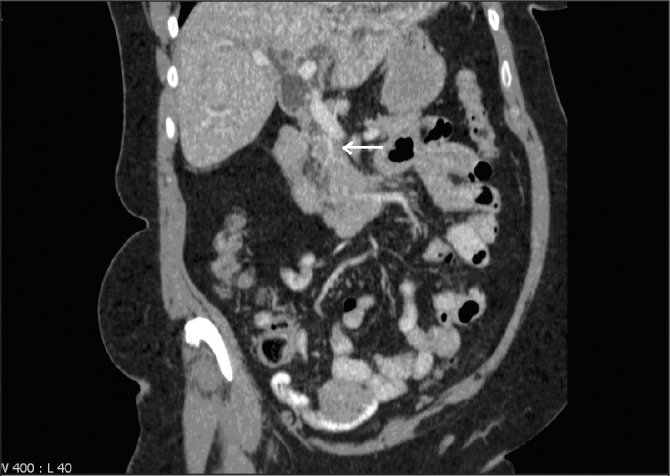
Coronal section of contrast-enhanced computed tomography of the abdomen and pelvis demonstrating a stricture at the mid-common bile duct (arrow)

A 70-year-old Caucasian woman with no significant alcohol consumption presented with a 4-week history of painless jaundice, anorexia and pruritus. She did not have any significant past medical history or family history of hereditary diseases. Clinical examination revealed she was jaundiced with no palpable masses or skin lesions. There was a right subcostal surgical scar from an open cholecystectomy performed two years previously. Laboratory analysis revealed deranged liver function tests suggestive of an obstructive presentation, with an elevated bilirubin level of 74μmol/l (normal range: 1– 21μmol/l) and alkaline phosphatase of 379iu/l (normal range: 40–130iu/l). Contrast enhanced computed tomography (CT) of the abdomen and pelvis identified a stricture at the mid-common bile duct (Fig 1) with no evidence of a mass lesion, vascular invasion or enlarged lymph nodes.

Following case discussion at the multidisciplinary team meeting, the patient underwent an exploratory laparotomy. At laparotomy, thickening of the mid-upper third of the common bile duct suspicious of malignancy was observed. There were no palpable lymph nodes or surrounding fibrosis suggestive of local invasion and no evidence of distant metastases. Intraoperative frozen sections were performed, both proximal and distal to the stricture to ensure margin clearance, which demonstrated characteristics of a benign lesion. The patient underwent excision of the extrahepatic bile duct and a Roux-en-Y hepaticojejunostomy.

**Figure 2:**
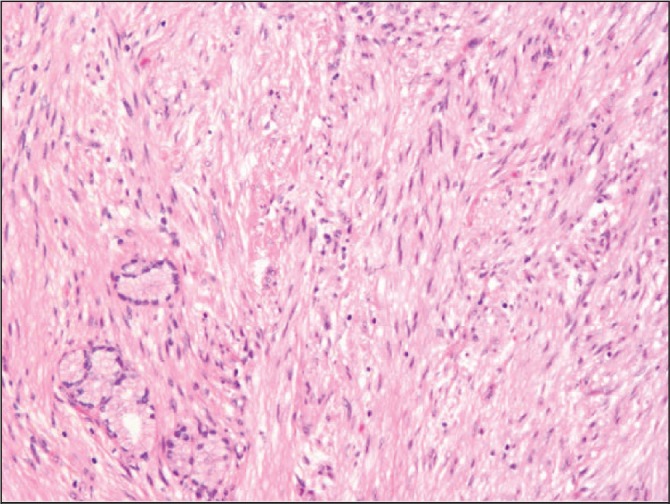
Haematoxylin and eosin stained section of the bile duct wall showing spindle cell proliferation (4× magnification)

**Figure 3:**
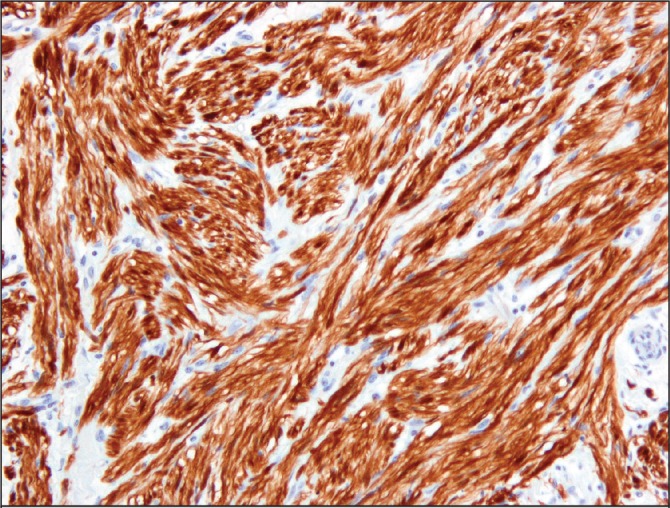
Positive staining of the lesion with S100, a marker for neurofilament protein (10× magnification)

The postoperative histological findings were consistent with a neurofibroma, with the presence of long spindle cells (Fig 2) and positive immunohistochemistry for S100, a marker for neurofilament protein (Fig 3). Following surgery, the patient made an uneventful recovery and was clinically well at the four-week follow-up appointment.

## Discussion

Neurofibromas are rare benign tumours arising from Schwann cells of nerve sheaths. Solitary neurofibromas may occur but they occur most frequently as part of neurofibromatosis, an autosomal dominant disease characterised by multiple tumours throughout the nervous system with or without skin lesions. Bile duct neurofibromas arise from the sympathetic and parasympathetic nerve fibres in the wall of the common bile duct and are extremely rare, with a limited number of cases reported in the literature. These tumours are characterised pathologically by the presence of long spindle cells, whose neurogenic properties are demonstrated by positive immunohistochemistry for S100, a marker for neurofilament protein.

The most common primary tumours of the biliary tract are cholangiocarcinomas, most of which are adenocarcinomas. Surgery is the only curative treatment with a 9–18% five-year survival rate for proximal bile duct lesions and a 20–30% five-year survival for distal lesions.^2^ Curative surgical resection is an option for only a minority of patients as the majority of patients present late when the lesion is unresectable. The five-year survival rate for inoperable disease is less than 5%.^3^ Benign tumours of the biliary tract, most commonly adenomas or papillomas, account for only 6% of all biliary tumours.^4^

In the absence of neurofibromatosis, almost all cases of neurofibromas of the common bile duct are secondary to operative trauma following a cholecystectomy,^5^ where traumatic rupture of the epineurium results in proliferation of the nerve fibres in the connective tissue surrounding the nerve. Solitary primary neurofibromas of the common bile duct may also occur, in which axonal proliferation is confined to the inside of the epineurium, and are thought to be the result of chronic inflammatory lesions, infection or foreign bodies such as biliary stones.

Symptomatic neurofibromas usually present with jaundice, with or without abdominal pain and/or fever. Such patients are investigated radiologically with CT as in this case, with or without endoscopic ultrasonography (EUS).^1^ The appearances of a homogenous mass or stricture are non-diagnostic in both imaging modalities and the use of EUS to investigate this patient would have not altered the subsequent management. The non-specific clinical and radiological features make it extremely difficult to differentiate between a neurofibroma and a malignant bile duct lesion preoperatively, and diagnosis is achieved only by histological examination.^1^ We emphasise the importance of intraoperative frozen section examination not only to ensure clear resection margins but also to rule out malignancy and avoid extensive surgical resection for benign disease, which could be associated with higher morbidity.

## Conclusions

Neurofibromas of the bile duct are rare, and the lack of specific clinical and radiological features makes preoperative diagnosis difficult. The diagnosis should be considered in patients presenting following previous bile duct trauma such as a cholecystectomy. An exploratory laparotomy should include the use of intraoperative frozen section to ensure clear margins and confirm benign disease, to enable the most appropriate surgical management to be instituted.

## References

[1] Ray S , Das K , Mridha AR , Khamrui S . Neurofibroma of the common bile duct: a rare cause of obstructive jaundice. Am J Surg 2011; 202: e1–e3 21741514 10.1016/j.amjsurg.2010.09.008

[2] Khan SA , Davidson BR , Goldin R *et al.* Guidelines for the diagnosis and treatment of cholangiocarcinoma: consensus document. Gut 2002; 51 Suppl 6: vi1–vi9 12376491 10.1136/gut.51.suppl_6.vi1 PMC1867742

[3] Farley DR , Weaver AL , Nagorney DM . ‘Natural history’ of unresected cholangiocarcinoma: patient outcome after noncurative intervention. Mayo Clin Proc 1995; 70: 425–429 7537346 10.4065/70.5.425

[4] Burhans R , Myers RT . Benign neoplasms of the extrahepatic biliary ducts. Am Surg 1971; 37: 161–166 5548431

[5] Peyré CG , Wakim M , Mateo R *et al.* Unusual cases of jaundice secondary to non-neoplastic bile duct obstruction. Am Surg 2004; 70: 620–624 15279187

